# A tarsus construct of a novel branched polyethylene with good elasticity for eyelid reconstruction *in vivo*

**DOI:** 10.1093/rb/rbaa001

**Published:** 2020-02-07

**Authors:** Peifang Xu, Xue Feng, Honghao Zheng, Zhongwei Feng, Zhisheng Fu, Changyou Gao, Juan Ye

**Affiliations:** r1 Department of Ophthalmology, The Second Affiliated Hospital of Zhejiang University, College of Medicine, Hangzhou, Zhejiang 310009, China; r2 MOE Key Laboratory of Macromolecular Synthesis and Functionalization, Department of Polymer Science and Engineering, Zhejiang University, Hangzhou 310027, China

**Keywords:** branched polyethylene, elasticity, scaffolds, fibrovascularization, eyelid reconstruction

## Abstract

Branched polyethylene (B-PE) elastomer was investigated for its potential medical application as a tarsus construct. The *in vitro* results showed that the B-PE and processed B-PE films or scaffolds did not exhibit noticeable cytotoxicity to the NIH3T3 fibroblasts and human vascular endothelial cells (ECs). The B-PE scaffolds with a pore size of 280–480 µm were prepared by using a gelatin porogen-leaching method. The porous scaffolds implanted subcutaneously in rats exhibited mild inflammatory response, collagen deposition and fast fibrovascularization, suggesting their good biocompatibility. Quantitative real-time PCR analysis showed low expression of pro-inflammatory genes and up-regulated expressions of collagen deposition and vascularization-related genes, validating the results of historical evaluation in a molecular level. The B-PE scaffolds and Medpor controls were transplanted in rabbits with eyelid defects. The B-PE scaffolds exhibited a similar elastic modulus and provided desirable repair effects with mild fibrous capsulation, less eyelid deformities, and were well integrated with the fibrovascular tissue compared with the Medpor controls.

## Introduction

Polyethylene (PE) can be synthesized by polymerization of ethylene in a number of ways. The way produced will affect its structure and thereby properties [1]. Many devices employed in different fields (industry, medicine, agriculture, mechanics, chemistry and transport etc.) use PE because of its controllable chemical and physical properties [2]. In medicine, PE is widely available in the construction of medical devices, ranging from simple devices to implants. Among the various categories of PE, high-density PE (HDPE) and ultra-high-molecular weight PE (UHMWPE) are most widely used in medicine [3]. HDPE has a high degree of crystalline structure and low degree of branching, thus resulting in strong intermolecular force and tensile strength [4]. The high-density porous PE (Medpor) composed of HDPE microspheres is used as alternative to autogenous graft for augmentation in many common sites such as orbital floor, cranium, and facial skeleton implant [5]. The high-molecular weight results in high toughness, excellent wear and chemical resistance, which allows UHMWPE to be used as the wear-bearing surface of hip and knee arthroplasty and total artificial joints [2]. However, they still have some disadvantages to be reckoned with, including their rigidity and sometimes they are palpable extra orally [5]. Hence, the HDPE and UHMWPE are considered as the ones of the best hard tissue substitutes, but have a restricted application in soft tissues.

Branching affects the crystallinity, which refers to the structural order in PE. A higher degree of branching in the polymer chain leads to lower crystallinity and randomly arranged molecules. The branched PE (B-PE) has a smaller viscosity, in the solution phase or in melts, thus increases its processability and elasticity [6]. The B-PE also has an improved solubility in organic solvent together with excellent film-forming ability [7]. In this study, the home-synthesized B-PE elastomer is also able to be made into a porous scaffold by a porogen-leaching method, which is very unique for this type of polymers. The improved polymer elasticity and scaffold porosity may make B-PE more preferable for soft tissue substitution.

Eyelid is a functional structure which is crucial to the integrity of the globe. The tarsus is a plate of tissue that stiffens the eyelid, making it an essential component of the eyelid’s function and appearance. Eyelid defect requiring reconstruction is commonly secondary to trauma, chemical injury or surgical excision of neoplasms. The eyelid would suffer from retraction or entropion, leading to corneal irritation and visual impairment, if no suitable substitute for the tarsal plate reconstruction is applied [8, 9]. Clinically, the tarsal substitute materials can be either autologous (contralateral eyelid [10], postauricular skin [11], hard-plate mucosa [12] and nasal septum [13]) or homologous (bank sclera, acellular dermal matrices [14] and aortic valve [15]). However, some disadvantages such as additional surgery, immunologic rejection and shrinkage and absorption have limited the application of human-derived grafts. Although the synthesized materials such as porous HDPE (Medpor), poly(3-hydroxybutyrate-co-3-hydroxyhexanoate) [16], and poly(propylene fumarate)-2-hydroxyethyl methacrylate copolymers [17] have been explored as tarsus substitutes, only the Medpor spacer has been used clinically. Tan *et al*. [18] reported a case series of 32 patients who underwent unilateral Medpor implantation, and gained a high level of patient acceptability in lid heightening and stabilization. However, there were complications such as implant exposure through the skin, unexplained pain, poor mobility and skin contour abnormalities in nearly 40% patients. Unlike Medpor, a linear highly compressed aliphatic hydrocarbon, B-PE possesses extensive branch-on-branch structures, and exhibits better elasticity and higher elongation [19]. Therefore, B-PE may have potential applications in making a mechanically optimized tarsus construct, in the premise of good biocompatibility.

In this study, a type of home-synthesized B-PE is fabricated into porous scaffolds by a porogen-leaching method, and is used as the tarsus construct. Its biocompatibility is assessed *in vitro* by using endothelial cells (ECs) and fibroblasts, and *in vivo* by subcutaneous implantation of in rats. The potential application of B-PE scaffold as a tarsal plate is assessed in eyelid reconstruction in a rabbit eyelid defect model *in vivo*.

## Materials and methods

### Preparation and characterization of B-PE films and scaffolds

The B-PE was synthesized according to literature by using (α-diimine) nickel as the catalyst [20]. The weight average molecular weight (*M*_w_) and polydispersity index of B-PE were 26.9 × 10^4^ g/mol and 3.6, respectively. It had a branch density of 29/1000 carbons determined by ^1^H-NMR, and a melting point and crystallinity of 121.5°C and 30.6% calculated by χ = ΔHm/ΔHm^o^ (ΔHm^o^ = 292.6 J/g), respectively.

The B-PE was dissolved in decalin (Sigma) to prepare a 5% (w/v) solution in an oil-bath at 100°C with mechanical agitation, which was used to prepare the B-PE films by a solution casting method. In brief, the B-PE solution was dropped onto a glass substrate and kept in a draught cupboard to obtain the polymer films. The B-PE scaffolds were fabricated using a gelatin porogen-leaching method as described previously [21]. Briefly, the sieved gelatin particles (280–480 μm) were added into glass molds, into which ethanol solution (v/v, 85%) was injected to immerse the particles. The top surface of the gelatin particles was pressed slightly to enable the gelatin particles connected with each other, and then the mold was kept in an oven at 40°C overnight. The template made of the connected gelatin particles was immersed into the B-PE solution under reduced pressure, allowing the B-PE solution to infiltrate into the template. After the solvent was evaporated, the gelatin particles were leached out by water to obtain the porous B-PE scaffolds.

The wettability of the films was characterized by measuring the water contact angle on the sample surface [22]. For this purpose, a drop of water was mounted on the films with a microsyringe. The contact angle was then obtained according to the droplet image. The morphology of the films and scaffolds were investigated under scanning electron microscopy (SEM, JEOL Ltd. JSM-5510LV). The porosity of the scaffolds was calculated by an ethanol displacement method [23]. Four parallel samples were used for this assay.

### *In vitro* cytotoxicity test assay

#### Cell line and culture

The NIH3T3 fibroblasts were obtained from American Type Culture Collection (ATCC Manassas, VA), and human vascular ECs were obtained from the Cell Bank of the Chinese Academy of Sciences (Shanghai, China). The cells were cultured in DMEM (Gibco) supplemented with 10% (v/v) fetal bovine serum (FBS), 1% (w/v) glutamine, 100 U/ml penicillin and 100 μg/ml streptomycin under standard conditions (5% CO_2_, 37°C).

#### Cell Counting Kit-8 (CCK-8) assay for cytotoxicity

The tests for the *in vitro* cytotoxicity were performed by using extract of B-PE, respectively. To prepare the liquid extract, the B-PE films were immersed in a serum-free culture medium at an approximate concentration of 0.1 g/ml at 37°C for 24 h. The extract was filtered by a 0.22 μm bacteria-retentive filter, and stored at −20°C before use. NIH3T3 fibroblasts and human vascular ECs were seeded into 96-well plates at 2 × 10^3^ cells per well. After being incubated for 24 h, the medium was removed and replaced with the extract supplemented with 10% (v/v) FBS. The normal culture medium was used as a positive control under the same conditions. Medium or extract was changed every 2 days. After 1, 4 and 7 days, the cell viability was measured by a CCK-8 assay. Briefly, 100 μl cell culture medium supplemented with 10 μl CCK-8 solution was added to each well, followed by incubation at 37°C for 2 h. The absorbance at 450 nm was measured by using a microplate reader (TECAN infinite 200PRO). Five parallel replicates at each time point were measured for this assay.

#### Cell viability and proliferation

B-PE films were placed into a 48-well plate and were sterilized with ultraviolet light for 30 min. NIH3T3 fibroblasts and human vascular ECs were seeded into the plates at a density of 1 × 10^4^ cells/cm^−2^, and were incubated in standard culture conditions for 24 h. The cell viability on the B-PE films was examined after 24 h seeding using Live/Dead staining, a two-color discrimination of living cells from dead cells. Living cells were stained with fluorescein diacetate (FDA), and dead cells were stained with propidium iodide. The fluorescent images were taken with a fluorescent microscope (IX81, Olympus). EdU Cell Proliferation Kit with Alexa Fluor 594 (Beyotime, Cat. No: C0078S) was used to visualize the proliferating cells. At 22-h post-seeding, 10 µM EdU was added into the medium. After another continuous culture of 2 h, the cells were fixed and stained for EdU uptake. Nuclei were counter-stained with Hoechst 33342. After an additional wash in PBS, the cells were observed under an inverted fluorescent microscope (DMI8, Leica).

### Mechanical test

The stress–strain curves of B-PE scaffolds, Medpor spacers and tarsal plates from New Zealand rabbits were measured by a universal mechanical testing instrument (Instron 5543A, USA). The tensile test was performed with a preload of 0.05 N before being strained at a set strain rate of 2 mm min^−1^ until mid-substance failure. The tensile modulus was calculated according to the slope of the linear part of the stress–strain curves at the initial strain. The elongation and breaking strength were calculated based on the intersection of a 0.2% offset line. The morphology of the samples after tensile test was characterized by SEM.

### Rat subcutaneous implantation

The animal experiments were performed according to the Guidelines of Animal Care and Use Committee, Zhejiang University. Six male Sprague–Dawley rats (two rats for each time point) weighing ∼120 g were anesthetized by intraperitoneal of a sodium pentobarbital solution (w/v, 3%) at a dosage of 1 ml/kg, and then shaved. A small incision (<1 cm) was made on the back of each rat. Blunt forceps were used to create a pocket in the subcutaneous space for scaffolds. One rat was implanted with four scaffolds. The scaffolds were harvested at days 7, 14 and 28, respectively. Gross view images of the harvested scaffolds were taken with a digital camera.

### Histological analysis and real-time PCR analysis

Two animals were sacrificed at determined time point by intraperitoneal injection of an overdose of sodium pentobarbital. The scaffolds and surrounding tissues were excised. Four scaffolds were fixed in 4% paraformaldehyde, embedded in paraffin and stained with Hematoxylin-eosin (H&E) and Masson staining for histology analysis. The thickness of the fibrous capsule was measured at ten different random locations per implant. Results from totally 10 such capsule areas were summarized, and the mean was calculated for each implant [24, 25]. The number of blood vessels in the vascular structures was counted in at four different sections. The slides were examined under a light microscope at 100× magnification, and six locations per section were selected randomly [26].

The expression of interleukins-1 (IL-1) and tumor necrosis factor (TNF-α), collagen type I (Col-I) and transforming growth factor-β (TGF-β), platelet endothelial cell adhesion molecule (PECAM-1, also termed as CD31) and vascular endothelial growth factor receptor 2 (VEGFR2, also termed as FLK-1) was analyzed at mRNA level for the evaluation of inflammation, collagen deposition and vascularization, repectively. The other four scaffolds were homogenized, and the total RNA was extracted using an Animal RNA Isolation Kit (Beyotime Biotechnology, China). The extracted RNA samples were reverse transcribed into complementary DNA (cDNA) using a PrimeScript RT Reagent Kit (Takara, China). The obtained cDNA was adopted as the template for subsequent PCR amplification. The quantitative real-time PCR (qRT-PCR) reactions were performed using the CFX96 system (Bio-Rad, USA) and the SYBR Premix EX Taq^TM^ kit (Takara, China). The primers used in real-time PCR studies are summarized in Supplementary Table S1.

### Implantation of porous B-PE scaffolds in eyelid defects

The B-PE porous scaffolds and Medpor implants (Porex Surgical Inc., College Park, GA) were cut into pieces with an average size of 6.0 × 3.0 × 0.7 mm, which were sterilized in 75% ethanol overnight and then balanced with sterile normal saline before use. The animal experiments were performed according to the Guidelines of Animal Care and Use Committee, Zhejiang University. Five male New Zealand white rabbits weighing 2.5–3.0 kg were randomized into two groups (five eyes for each group). Surgeries were performed under general anesthesia using pentobarbital sodium (30 mg/kg), followed by 1% lidocaine for local anesthesia as reported by our group previously [17]. A 3-0 silk traction suture was placed through the central portion of the upper eyelid to evert the upper lid and expose the palpebral conjunctiva. The palpebral conjunctiva was divided from the tarsal plate. The tarsal defects of 6.0 × 3.0 mm was carefully created 2-mm away from the palpebral margin. The scaffolds were trimmed to fit the wound beds and placed into the defect sites. The grafts were then sutured to the tarsal stumps by 7-0 polyglactin. Last, the palpebral conjunctiva was closed with 8-0 biodegradable sutures. Tobramycin and dexamethasone ointments were used immediately after surgery. For histological evaluation, the eyelid samples with implants were harvested at fourth week and were fixed in formalin and embedded in paraffin, which were sectioned and stained with H&E and Masson staining.

### Statistical analysis

Results are reported as mean ± SD. Statistical analysis was performed by the Student’s test analysis in Origin 9.1 software. The significance level was set as *P *<* *0.05.

## Results

### Characterization of B-PE materials

**Figure 1:**
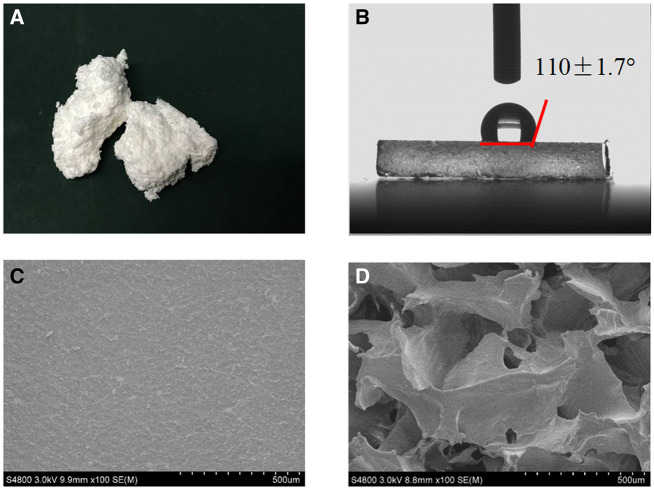
Digital images of B-PE appearance (**A**) and water droplet showing wettability (**B**). SEM images of B-PE film (**C**) and scaffold (**D**)

The B-PE was a foam-like elastomer in appearance and had a hydrophobic surface with an average contact angle of 110 ± 1.73° (Fig. 1A and B). The obtained scaffolds showed a porous structure with high porosity (90.26 ± 2.08%) and interconnectivity. The pore size was 280–480 μm, controlled by the template of sieved gelatin particles.

### Cytotoxicity of the B-PE materials

**Figure 2:**
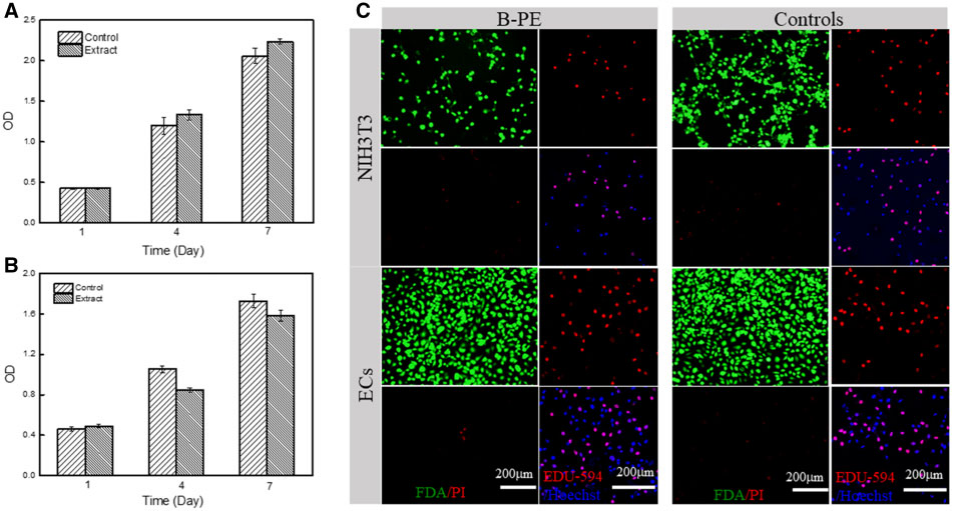
Cytotoxicity of B-PE material. The viability of (**A**) NIH3T3 fibroblasts and (**B**) human vascular ECs as a function of culture time. Cells were cultured in DMEM extract from B-PE material and the normal DMEM medium, respectively. Error bars represent means ± SD (*n* = 5). (**C**) Viability and proliferation of NIH3T3 fibroblasts and human vascular ECs on B-PE films. Living cells were stained by FDA, and dead cells were stained by propidium iodide. EdU uptake was stained by Alexa Fluor 594, and nucleus was stained by Hoechst 33342. Cells were cultured on B-PE films and TCPS control, respectively

CCK-8 tests were performed to determine whether B-PE extract was cytotoxic to NIH3T3 fibroblasts and human vascular ECs *in vitro*. The extract did not induce significant changes in cell viability compared with negative controls (fresh medium with no extracts; Fig. 2A and B). Cytotoxic and bioactive effects of B-PE were also determined when the polymer films were in direct contact with NIH3T3 fibroblasts and human vascular ECs *in vitro* (Fig. 2C). Live/dead assay indicated that the B-PE films did not induce significant increase in apoptosis compared with tissue culture polystyrene (TCPS). Human vascular ECs adhered, spread and proliferated on the surface of B-PE films after 24 h culture, which were nearly indistinguishable from the cells grown on TCPS. The proliferating cells were determined by EdU staining. However, the NIH3T3 fibroblasts showed a poorer initial adhesion on the B-PE films than on TCPS. After 7-day culture, the human vascular ECs proliferated and formed an endothelium lining or paving at the surfaces and pore walls of the B-PE scaffolds (Supplementary Fig. S1).

### Biomechanical properties of B-PE scaffolds

**Figure 3:**
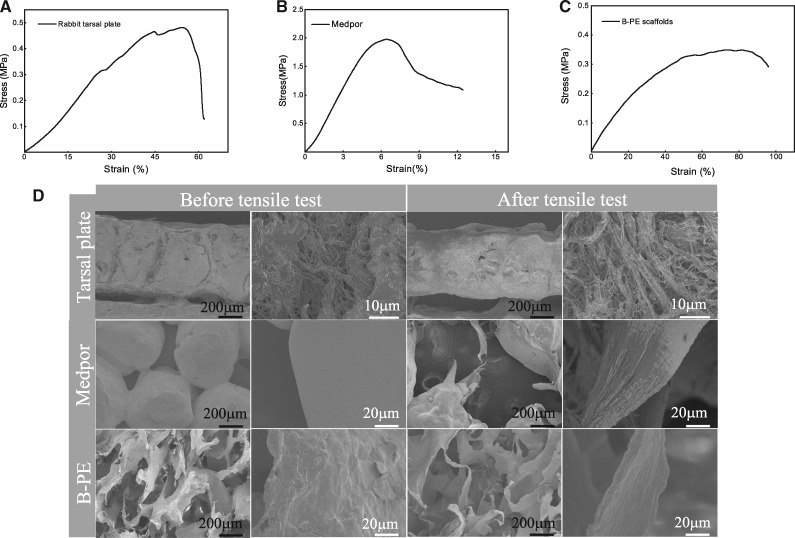
Tensile stress–strain curves (**A–C**) and (**D**) morphology of rabbit tarsal plates, Medpor spacers and B-PE scaffolds before and after tensile tests

The uniaxial tensile tests were performed on samples of rabbit tarsal plates, Medpor spacers and B-PE scaffolds to measure their ultimate tensile strength, mean failure strain and mean elastic modulus (Table 1). Of the tarsus samples tested, the mean elastic modulus was 0.92 ± 0.05 MPa with an extensibility of 50.8 ± 10.4% (Fig. 3A). The Medpor eyelid spacers, used for lid heightening and stabilization in clinical, displayed very different mechanical properties, with an average elastic modulus of 30.2 ± 4.5 MPa and total elongation to failure limited in about 7% (Fig. 3B). In contrast to the Medpor spacers, the B-PE scaffolds exhibited comparable mechanical properties with the natural eyelid tarsal plates. The B-PE scaffolds displayed a gradually increasing strain after applied stress, with an elastic modulus of ∼0.83 ± 0.1 MPa and a mean failure strain of 76.2 ± 2.8% (Fig. 3C). The change of morphology of samples before and after the tensile tests is shown in SEM images (Fig. 3D). The meibomian glands were embedded within the natural tarsal plate regularly, surrounded with extracellular matrix. However, after being drawn, fiber thinning and disorder structure of meibomian glands were observed. In the Medpor spacers, larger pores appeared due to fractures at the adhesive interface of HDPE microspheres. The B-PE scaffolds were stretched along the tensile direction. When reaching ultimate strength, part of the scaffolds was torn with longitudinal local direction.

**Table 1:** Mechanical properties of rabbit tarsal plate, Medpor and B-PE scaffolds

Sample	Tensile modulus (MPa)	Tensile strength (MPa)	Elongation (%)
Rabbit tarsal	0.92 ± 0.05	0.48 ± 0.1	50.8 ± 10.4
Medpor	30.2 ± 4.5	1.85 ± 0.3	7.0 ± 1.1
B-PE scaffolds	0.83 ± 0.1	0.30 ± 0.04	76.2 ± 2.8

### *In vivo* biocompatibility of B-PE scaffolds

**Figure 4:**
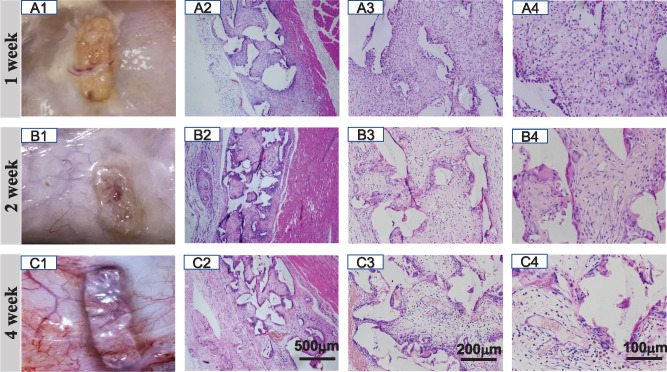
*In vivo* biocompatibility evaluation of B-PE scaffolds following subcutaneous implantation in rats. Gross view (**A1–C1**) and H&E staining sections (**A2–4, B2–4** and **C2–4**) after being implanted for (A) 1, (B) 2 and (C) 4 weeks, respectively. The B-PE scaffolds could be recognized as a white porous structure with tissue in growth, collagen deposition and neovascularization

To clarify the *in vivo* behaviors of B-PE scaffolds such as inflammation, fibrotic capsule and vascularization, the scaffolds were implanted subcutaneously in rats. After 1, 2 and 4 weeks implantation, the scaffolds were enucleated and the tissues around the scaffolds were carefully harvested. All specimens were observed visually (Fig. 4A1, B1 and C1). No tissue edema, subcutaneous hemorrhage or sever inflammation was found in any implant. After implantation, subcutaneous tissue grew around the implants gradually, and there appeared much richer vascular anastomoses at the fourth week than at the first and second week post-implantation. Thus, the scaffolds did not show obvious toxicity, and integrated well with their surrounding tissues.

### Histological analysis and gene expression in the process of implantation

As shown in Fig. 4, from 1 to 4 weeks after implantation, the fibrovascular tissues invaded into the scaffolds. Obviously, tissues grew into the scaffolds very rapidly, and reached the center of the scaffolds within 1 week (Fig. 4A2–4). The large conglomerates of inflammatory cells were infiltrated in and around the implants at the first week, which mostly were lymphocytes and macrophages. Several newly formed small blood vessels were also observed. From the second to fourth week, the tissues were dominated by fibroblasts, and foreign body giant cells could be found along the interface of implants and host tissues. Larger and more mature blood vessels and collagen fibrils were observed throughout the whole area of the implants (Fig. 4B and C).

**Figure 5:**
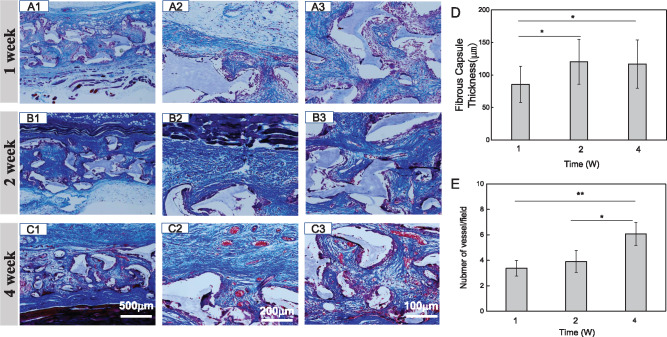
Masson staining sections of B-PE scaffolds for quantification of visualization and collagen deposition after being implanted for (**A**) 1, (**B**) 2 and (**C**) 4 weeks, respectively. The fibrous capsule thickness (**D**) and numbers of newly formed blood vessels (**E**) in tissue sections of B-PE scaffolds

The process of collagen deposition and fibrous capsules was also characterized by H&E staining and confirmed by Masson staining (Fig. 5A–C). There were more mature and dense collagenous fibers at the second and fourth week compared with the first week. The thickness of the peri-implant capsules at the first, second and fourth week was 85.6 ± 28, 120 ± 34.6 and 116.8 ± 37 μm, respectively (Fig. 5D). The peri-implant capsules were thickened significantly at the second week compared with the first week, whereas there was no significant difference in the fibrous capsule thickness between the second and fourth week. Figure 5E represents the time-dependent changes in the number of blood vessels in the implants. The numbers of blood vessels in the infiltrated tissue increased significantly with the implantation time.

**Figure 6:**
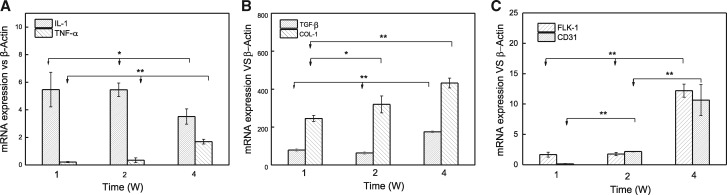
Expression of (**A**) inflammatory factors of IL-1 and TNF-α, (**B**) collagen deposition and (**C**) vascularization-related genes in the infiltrated tissues post-implantation for 1, 2 and 4 weeks. *P < 0.05, **P < 0.01

To understand the molecular mechanisms of local effects after implantation, the expression of genes closely related to inflammation response (Fig. 6A for IL-1 and TNF-α), collagen deposition (Fig. 6B for collagen Type I and TGF-β) and vascularization *in vivo* (Fig. 6C for CD31 and Flk-1) was quantified. The gene expression of inflammation-related target genes IL-1 and TNF-α was relatively low for all the implantation time (Fig. 6A). Moreover, the expression of IL-1 decreased at the fourth week, indicating a minor inflammation response. Although the expression of TNF-α increased at the fourth week, it was still at a low expression level. The deposition of collagen is a direct evaluation of biomaterial-mediated fibrotic responses. At the gene level, the expression of Col-I and TGF-β increased significantly along with time prolongation. This result was in concordance with the result of Masson staining. Flk-1 has high affinitive VEGF binding domains and is regarded as a major regulator of vasculogenesis and angiogenesis [27]. Platelet-EC adhesion molecule-1/CD31 is a constituent of the endothelial intercellular junction, and plays an important role in the adhesion cascade between ECs during angiogenesis [28]. Therefore, the gene expression of CD31 and Flk-1 was evaluated as markers of neo-vessel formation, which were enhanced along with time prolongation and became largest at the fourth week. These data indicate a better vascularization with implantation time prolongation.

### *In situ* implantation of B-PE scaffolds in eyelid defects

**Figure 7:**
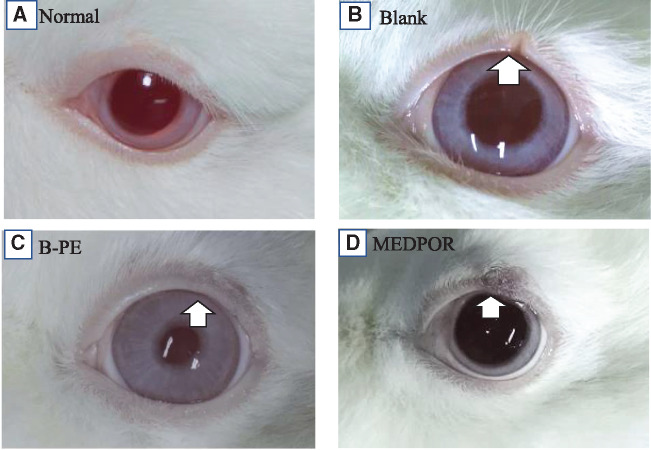
Post-surgery appearance at the fourth week. (**A**) Normal and (**B**) blank control. B-PE scaffolds group (**C**) showed well eyelid repair and Medpor spacers group (**D**) showed a hard hyperplasia and hyper pigmentation. The white arrows point to the defect sites

The porous B-PE scaffolds were chosen for the further *in situ* implantation to repair rabbit eyelid defects, and the results were compared with the Medpor spacers. Histological evaluation was carried out 4 weeks after implantation. After 4 weeks, severe contraction of tarsal defects could be found in the blank control group (Fig. 7B). The operated eyes showed good wound healing in the B-PE group (Fig. 7C) without noticeable contraction, rejection, necrosis or inflammation. In contrast, a hard hyperplasia with pigmentation was formed in the Medpor spacers group (Fig. 7D). One operated eye even showed material exposure without obvious local inflammation, indicating that the Medpor implants were not well tolerated by the host tissues (Supplementary Fig. S2D).

**Figure 8:**
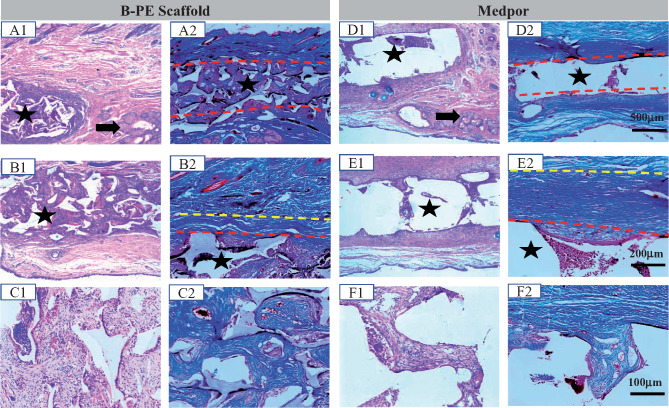
H&E staining (**A1–F1**) and Masson staining sections of (**A2−F2**) of (**A–C**) B-PE and (**D–F**) Medpor implants in rabbit eyelid defects at the fourth week. Asterisks represent scaffolds. Black arrows point to the normal meibomian glands. Red lines represent interface of tissues and materials. Tissues between the red and yellow lines are defined as the fibrous capsules

Histologically, the tissue response to the B-PE scaffolds presented abundant connective tissue infiltration (Fig. 8A–C and Supplementary Fig. S3), with ample vascular in growth and randomly organized collagenous fibers. At the fourth week, the degree of inflammatory response was mild as small conglomerates of inflammatory cells presented occasionally. The developed fibrovascular tissue in the pores of scaffolds was dominated by macrophages and fibroblasts. Foreign body giant cells presented at the material–host interface. However, the tissue response in the Medpor group turned out very different (Fig. 8D–F and Supplementary Fig. S2). A scar-like tissue was formed, which exhibited nodular structure composed of fibroblastic cells and regularly arranged collagenous fibers. The Medpor spacers were surrounded by thick fibrous capsules, and showed tissue infiltration deficiency. Masson staining confirmed that the Medpor spacers induced a severe fibrous encapsulation.

## Discussion

It is of paramount importance that any material used as an implant biomaterial has no untoward effects. The material should be non-toxic to surrounding cells and integrated well with host tissues [29]. Our data show that the novel B-PE elastomer is suitable to be used as a basic material for substitute of tarsal plate. It showed non-cytotoxicity *in vitro* and adequate fibrovascular infiltration *in vivo*, and could support and stabilize eyelid in eyelid defects.

The interactions between cells and synthetic biomaterials are of the highest importance, because these materials are the basic components of prostheses or implants designed to restore support and maintain organ and tissue functions [30]. Fibroblasts and ECs are major cells involved in the fibrous capsule formation and vascularization process upon biomaterial implantation. Therefore, *in vitro* culture of these two types of cells would be substantially useful for biocompatibility tests of B-PE for potential medical applications. Indeed, the *in vitro* cell culture system may serve as a first screening step for biocompatibility before *in vivo* evaluation [31]. The evaluation of biocompatibility was performed by exposing NIH3T3 fibroblasts and human vascular ECs to B-PE films or their extracts. The use of extract evaluates the effects of possible toxic contaminants that may readily be extracted from the B-PE. This stage is particularly important because biomaterials applied *in vivo* may be exposed to blood and tissue fluid, which act as the extraction vehicle. Cell viability and proliferation of NIH3T3 fibroblasts and human vascular ECs cultured with B-PE extract were not significantly different from those cultured with fresh medium (Fig. 2A and B). Direct exposure of the biomaterials to the target cells has been widely used in evaluation of cell–biomaterial interactions, which simulates *in vivo* conditions of direct interactions of host cells and tissues with implanted biomaterials. The direct contact assay confirmed that the B-PE was nearly non-cytotoxic and could support the adhesion and proliferation of human vascular ECs. However, the NIH3T3 fibroblasts demonstrated a relatively poorer adhesion and spreading on the B-PE film, probably due to its hydrophobic surface. Moreover, the NIH3T3 fibroblasts may be relatively more sensitive to surface wettability. The poor adhesion and spreading of cells could be improved by increasing the wettability of the material surface [32]. We speculate that the different cellular behaviors may be favorable to induce a moderate fibrous capsule and adequate vascularization *in vivo*.

Implantation of biomaterials triggers a series of host reaction at the injury site including material-tissue interactions, acute and chronic inflammation, foreign body reaction, fibrous capsule and vascularization [33]. After implantation, a layer of nonspecific protein immediately adsorbs onto the biomaterial surface [34], followed with the recruiting of defensive cells. The defensive cells primarily include cells of innate and adaptive immunity, such as neutrophils, macrophages, lymphocytes etc. The existence of these cells is based on the need to repel and remove injurious external agents [35]. Nearly nontoxicity *in vitro*, the B-PE may probably be biocompatible as other categories of PE such as Medpor and low-density PE (LDPE), which were approved by the Food and Drug Administration for medical use. A subcutaneous implantation was undertaken in rats to identify *in vivo* events occurring at the tissue/scaffold interface. As shown in this study, the B-PE exhibited a typical tissue-healing sequence, characterized by a mild inflammatory reaction in the early stage, followed by a more discrete chronic phase of inflammation, similar to LDPE and polydimethylsiloxane [36]. Though non-degradable, the B-PE itself is chemically inert, which may have no major effect on host physiological processes, and does not activate an unfavorable foreign body response.

The pore size, pore interconnectivity [37] and porosity [38] are crucial parameters for porous biomaterial scaffolds, which have a great impact on cell migration, tissue integration and grafts success. The high pore interconnectivity [37] and a pore size >300 μm [23, 39] are favorable for capillary ingrowth. In this study, 280–480 μm gelatin particles were used as the porogens to prepare the B-PE porous scaffolds, which had a high porosity and well-interconnected pores. These properties resulted in a good integration of B-PE implant with host tissue. Fast tissue invasion was expected at the first week. The pores were filled with new connective tissue featured with loose extracellular matrix, delicate capillaries, proliferation of fibroblasts and infiltration of inflammatory cells (Fig. 4). In the process of wound healing, the early inflammatory response and secreted cytokines could activate angiogenesis [40]. Then, TGF-β-mediated fibrosis is required for a transition from granulation tissue to mature tissue [41]. From the molecular biology level to histological observation, the quality of collagen deposition increased (Fig. 5B1–3), followed with collagen fibers remodeling and orientation. At the fourth week, large and mature blood vessels could be found in the loose collagen fibers with random alignment rather than linear scarring, indicating a satisfactory healing of the scaffold implantation (Fig. 5C1–3). Blood vessels that formed in the regenerated tissues are also likely to be involved in the maintenance of homeostasis, which provide oxygen and nutrients, and remove waste products [42]. It is only through local tissue response that a dynamic interaction occurring between activated inflammatory cells, secreted enzymes and cytokines can stimulate fibroblast proliferation and collagen production, as well as angiogenesis, in a chain of events leading to the encapsulation and satisfactory healing of a prosthesis or implant [36]. Hence, the porous B-PE scaffolds within rat dorsal soft tissue constituted a biocompatible system.

The ideal biological tarsal substitutes should have appropriate properties including thickness, strength and flexibility that mimic a natural tarsal plate, in addition to good tissue compatibility [43]. Tissue engineering, allowing for flexibility of structure and design, offers a promising technique to produce a potential tarsal substitute biomechanically and structurally similar to natural human tarsus. High porosity and interconnectivity have been shown to greatly affect the macroscopic mechanical properties of scaffolds [44] and benefit the developing of cells and tissues in the engineered tarsus construct [45]. In this study, the B-PE elastomer was reshaped into a porous scaffold. The porous B-PE scaffolds displayed an ultimate tensile strength of 0.30 ± 0.04 MPa and a maximum strain of ∼76.2%, which in a way match the natural tarsal plate for the overlying soft tissues. Therefore, the biomechanical properties of the B-PE scaffold may enable it to act as a ‘skeletal element’ for the eyelid stabilization. It has been suggested that material-related properties such as surface chemical properties, mechanical properties and topography might influence soft tissue response, fibrous capsule thickness and quality [24, 46]. Recent studies by Wipff *et al*. [47] demonstrated that fibroblast activation of TGF-β increases on rigid substrates, leading to greater myofibroblast differentiation. Histologically, the implantation of rigid Medpor spacers in rabbit eyelid defect leads to a thicker fibrous capsule, partially bridging through the micropores as reported [48], while the B-PE scaffolds showed better eyelid repair, without obvious scar contraction, rejection or unfavorable local tissue reaction. We hypothesize that the mismatched rigidity of Medpor to eyelid activates fibroblast more easily, leading to a higher level of TGF-β-mediated epithelial-to-mesenchymal transitions and subsequently fibrous capsule. A thicker capsule may be one kind of compensation mechanisms to moderate mechanical shear [24]. Excessive fibrous capsule would create a contractile force originating, and cause capsular contracture around the implant, making the capsule hardened [49]. That helps to explain why some patients are suffered from unexplained pain and poor lid motility when treated with Medpor for spacer lid stabilization[18].

Natural tarsal plate could be defined as a unique tissue with some of the features of both dense fibrous connective tissue and cartilage [50]. Numerous collagen fibers are present throughout the tarsal plate, within which are orderly distributed meibomian glands and a number of small blood vessels. In this study, the B-PE scaffolds acted as a skeletal element and enabled sufficient fibrovascular tissue to infiltrate into the scaffolds, leading to the excellent incorporation and stabilization of implant [51]. Meanwhile, the porous structure may have helped interrupt the continuity and linear orientation of collagen formation [52]. We would therefore expect that the B-PE scaffolds sufficiently integrated with the randomly organized collagen fibers would exhibit biomimetic mechanical properties of natural tarsal plate. Sequentially, the reconstructed eyelid would be elastic enough to be mold to the shape of globe, and not too stiff to cause discomfort. According to this study, the B-PE scaffold would be a potential engineered tarsus construct for structure restoration of eyelid abnormity.

## Conclusion

The B-PE material in the form of films or scaffolds was obtained and tested. The gelatin porogen-leaching method was a proper way to obtain the porous B-PE scaffolds with interconnected pores. *In vitro* cytotoxicity tests by both extract test and direct contact test showed that the B-PE exhibited no obvious cytotoxicity to NIH3T3 fibroblasts and human vascular ECs. Furthermore, accompanied by a moderate inflammatory response, the rapid fibrovascular engraftment of scaffolds occurred within 1 week *in vivo*, which is regarded as key factor for its successful tissue integration. After grafted in rabbit tarsal plate full-thickness defects, the B-PE scaffolds provided a better eyelid reconstruction compared with the Medpor tarsal spacers. Therefore, these results have demonstrated the potential of B-PE scaffold to be used as a tarsal plate substitute. 

## Supplementary Material

rbaa001_Supplementary_Data
