# Supplementary material for: A tarsus construct of a novel branched polyethylene with good elasticity for eyelid reconstruction in vivo
Source: Regen Biomater. 2020 Feb 7;7(3):259–69. doi: 10.1093/rb/rbaa001 (PMC7266665; doi:10.1093/rb/rbaa001)
Supplement: rbaa001_Supplementary_Data [file rbaa001_supplementary_data.pdf]

Supplemental Materials

A tarsus construct of a novel branched polyethylene with good elasticity for eyelid reconstruction *in vivo*

Peifang Xu[a], Xue Feng[b], Honghao Zheng [b], Zhongwei Feng[b], Zhisheng Fu[*b], Changyou Gao[*b] and Juan Ye[*a]

[a] Department of Ophthalmology, the Second Affiliated Hospital of Zhejiang University, College of Medicine, Hangzhou, Zhejiang, 310009, China.

E-mail: yejuan@zju.edu.cn (J. Y)

[b] MOE Key Laboratory of Macromolecular Synthesis and Functionalization, Department of Polymer Science and Engineering, Zhejiang University, Hangzhou, 310027, China.

E-mail: fuzs@zju.edu.cn (Z.F), cygao@zju.edu.cn (C.G.)

Table S1 Primers for real-time PCR experiments

Gene	Primers	
β-actin	F	5’-CCCATCTATGAGGGTTACGC-3’
	R	5’-TTTAATGTCACGCACGATTTC-3’
TGF-β	F	5’-ATTCCTGGCGTTACCTTGG-3’
	R	5’-AGCCCTGTATTCCGTTCTCT-3’
Col-Ⅰ	F	5’-CAGTCGATTCACCTACAGCACG -3’
	R	5’-GGGATGGAGGGAGTTTACACG-3’
IL-1	F	5’-CATCTTTGAAGAAGAGCCCG-3’
	R	5’-TGTTCGTTGCTGTTTTAGGG-3’
TNF-α	F	5’-CTATGTGCTCCTCACCCACA-3’
	R	5’-ATGGACCCTCCTCAGAAGGT-3’
CD31	F	5’-GCCGGTCACCCTTCTCCAAC-3’
	R	5’-CAGAGTCTGGCACTGCTTT-3’
vWF	R	5’- GTCGGAAGAGGAAGTGGACATT -3’
	F	5’- GGGCACACGCATGCGCTCTGTA -3’
FLK-1	F	5’- GTCAAGTGGCGACGGTAAAGG -3’
	R	5’- GTGTGGCAAGACAGAAGTGGAGTT -3’

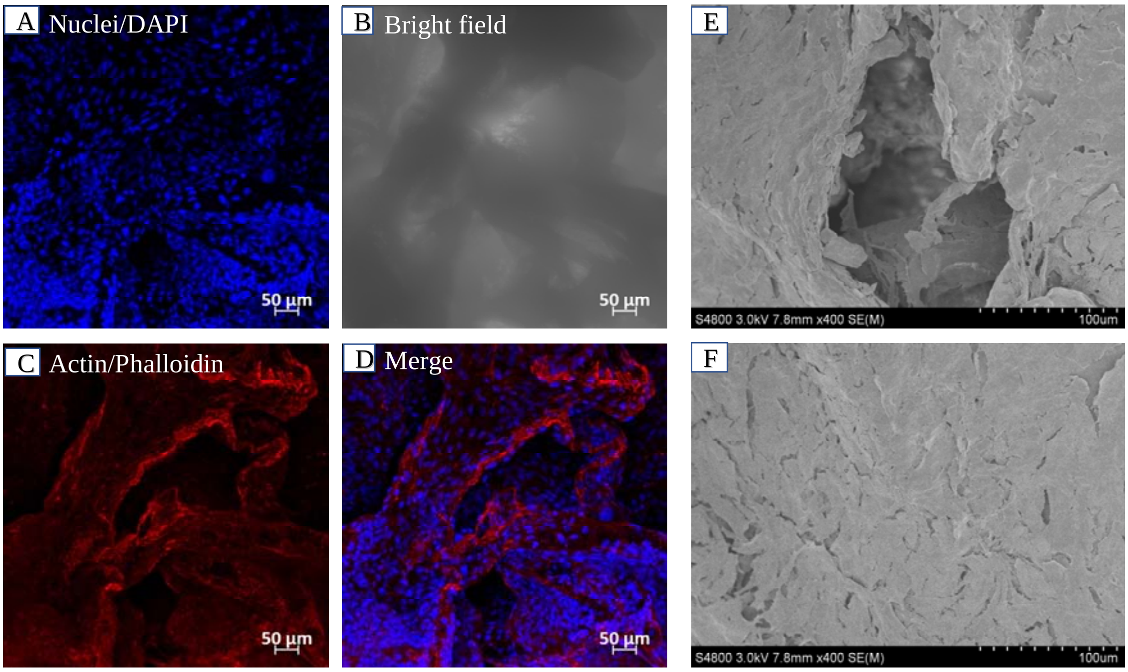

Fig. S1 CLSM images (A-D) and SEM images (E, F) showing the morphology and distribution of human vascular ECs inside the B-PE scaffolds after 7 d culture *in vitro*. The nuclei (A) and actin (B) were stained with DAPI (blue) and rhodamine phalloidin (red), respectively. (B) is a bright field image, and (D) is the merged images of (A) and (C).

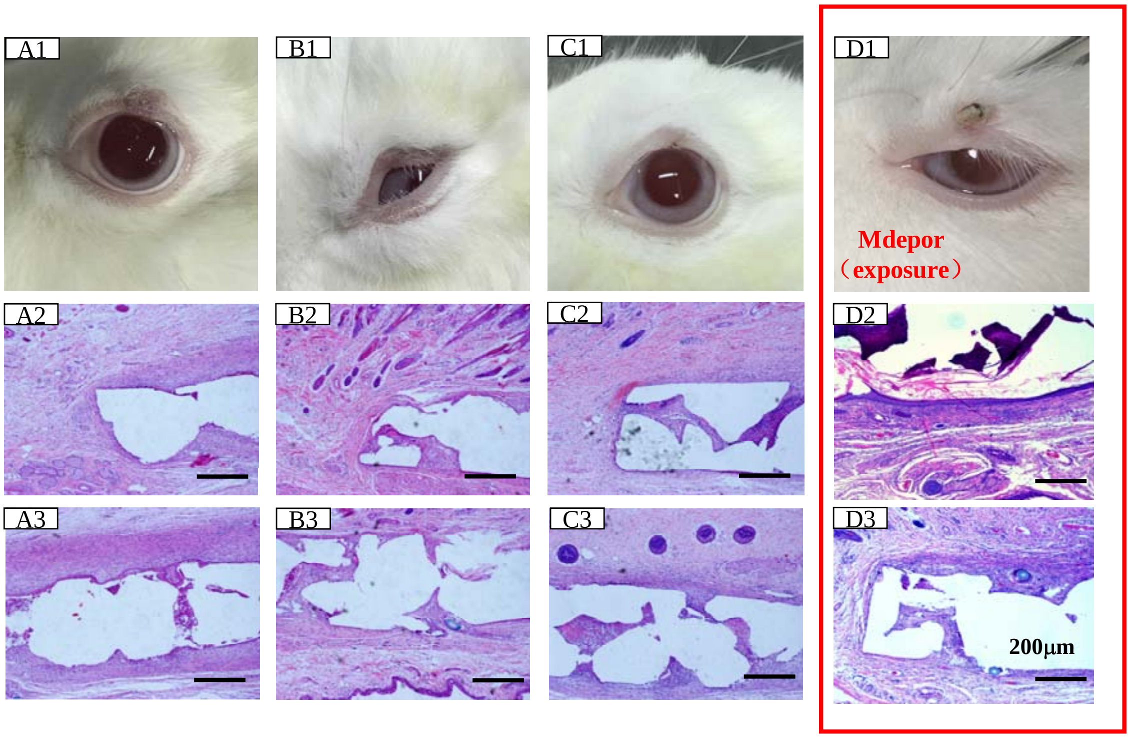

Fig. S2 Post-surgery appearance and H&E staining of sections of parallel samples after being treated with Medpor spacers for tarsal plate repair. One rabbit (D1-D3) showed an exposure of Medpor spacer.

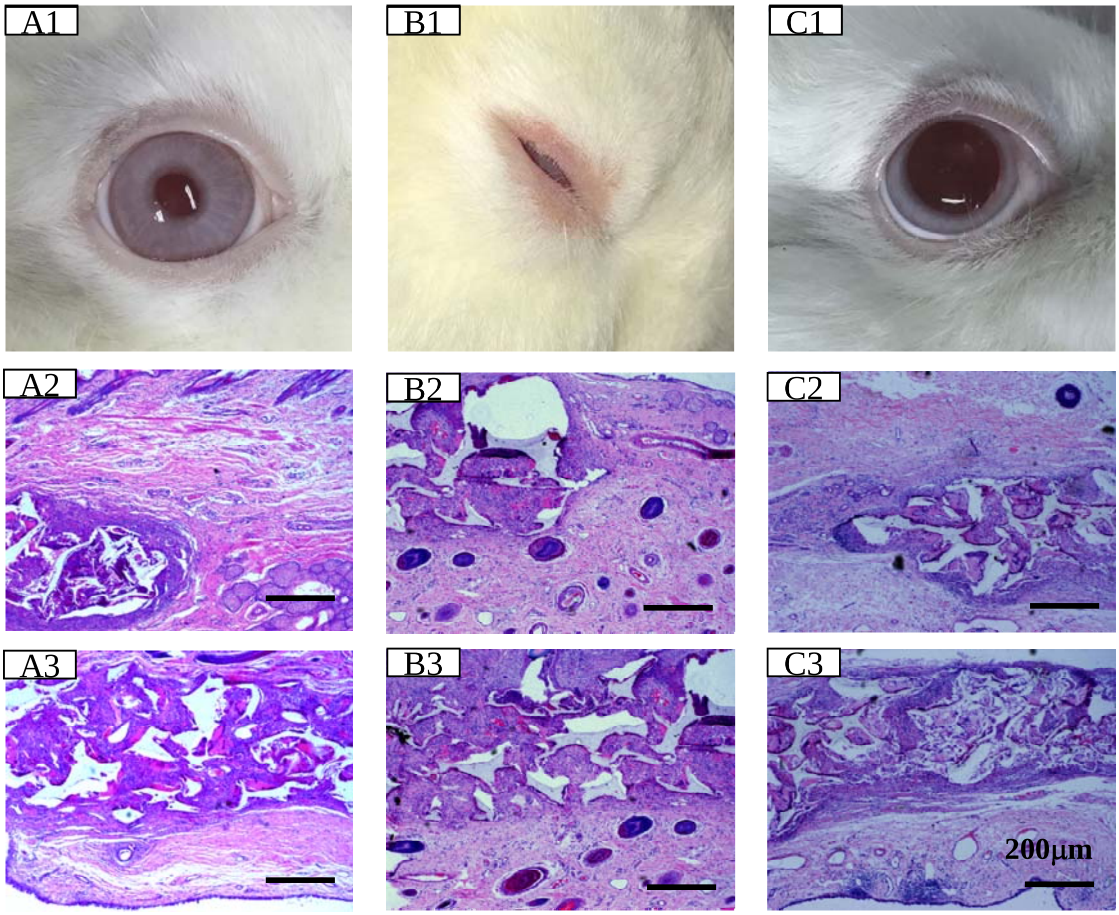

Fig. S3 Post-surgery appearance and H&E staining of sections of parallel samples after being treated with B-PE scaffolds for tarsal plate repair.
